# Supplementary material for: The prevalence of fimA genotypes of Porphyromonas gingivalis in patients with chronic periodontitis: A meta-analysis
Source: PLoS One. 2020 Oct 28;15(10):e0240251. doi: 10.1371/journal.pone.0240251 (PMC7592798; doi:10.1371/journal.pone.0240251)
Supplement: Attachment — Submitted filename: Response to Reviewers.docx [file pone.0240251.s002.docx]

Dear Editor, ReviewerS
RE: Manuscript PONE-D-20-06291

We would like to thank PLOS ONE for giving us the opportunity to revise our manuscript.
We thank the reviewers for their careful read and thoughtful comments on previous draft. We have carefully taken their comments into consideration in preparing our revision, which has resulted in a paper that is clearer, more compelling, and broader. The following summarizes how we responded to reviewer comments.
Below is our response to their comments.
Thanks for all the help.

Best wishes,

Haini Wang
The first Author

Revision -author' response

1.Is the manuscript technically sound, and do the data support the conclusions?

The manuscript must describe a technically sound piece of scientific research with data that supports the conclusions. Experiments must have been conducted rigorously, with appropriate controls, replication, and sample sizes. The conclusions must be drawn appropriately based on the data presented.

Reply: The text has been changed according to the reviewer comments.

4. Is the manuscript presented in an intelligible fashion and written in standard English?

PLOS ONE does not copyedit accepted manuscripts, so the language in submitted articles must be clear, correct, and unambiguous.Any typographical or grammatical errors should be corrected at revision, so please note any specific errors here.

Reply: The text has been changed according to the reviewer comments.

Reviewer #1:
- English remains a concern, and authors are strongly encouraged to thoroughly revise their draft. Please check grammar and spelling. Please seek some help from a native speaker, to revise, and to facilitate reading.

Reply: The text has been changed according to the reviewer comments.

Abstract
- "Therefore, the two genotypes of Porphyromonasgingivalis appear to contribute to the pathogenesis and progress of periodontal disease, one of the main risk factors of periodontitis." How can you say this? Please provide a sound conclusion, only based on your outcome.

Reply: The text has been changed according to the reviewer comments.

Intro
- "Plaque is the initiating factor of periodontal disease." Please note that "plaque" is a dental term. Use "biofilm" instead, and add a definition.
-Please revise writing, see: "aggregatibacte,Actinomycetemcomitans,Treponemadenticola, Prevotella nigrescens,and Pusobacterium nucleatum". Incorrect spelling is not acceptable with a scientific paper.
- Please elaborate your aims, and give your objectives.
- Deduce a sound and indisputable null hypothesis from your foregoing elaboration.

Reply: The text has been changed according to the reviewer comments.

Meths
- Please expand this section by given more details.

Reply: The text has been changed according to the reviewer comments.

Results
- Again, please expand this section, to facilitate reading.
- Please note that it is your task to guide the readers. Do not leave the latter alone with your graphs. Provide a neutral explanation.

Reply: The text has been changed according to the reviewer comments.

Disc
- The same with this section. Please note that your Discussion has not been convincingly elaborated.

Reply: The text has been changed according to the reviewer comments.

Concl
- "Therefore, the two genotypes of Porphyromonas gingivalis are related to the pathogenesis and progress of periodontal disease." Please see comments given above

Reply: The text has been changed according to the reviewer comments.

Refs
- Following the Authors' Guidelines is strongly recommended.

Reply: The text has been changed according to the reviewer comments.

Reviewer #2: Review Comments to the Author

"Nice paper"

Reply: Thank you.

Reviewer #3:

Please check word spacing, organism names and syntax errors in the manuscript. No other major corrections is noted. All the relevant data has been mentioned and the manuscript written in intelligiblefashion.

Reply: The text has been changed according to the reviewer comments.

6. PLOS authors have the option to publish the peer review history of their article ([what does this mean?](https://journals.plos.org/plosone/s/editorial-and-peer-review-process#loc-peer-review-history)). If published, this will include your full peer review and any attached files.

If you choose “no”, your identity will remain anonymous but your review may still be made public.

**Do you want your identity to be public for this peer review?** For information about this choice, including consent withdrawal, please see our [Privacy Policy](https://www.plos.org/privacy-policy).

Reply: I have been changed according to the reviewer comments.

Thank you!
