# Supplementary material for: The prevalence of fimA genotypes of Porphyromonas gingivalis in patients with chronic periodontitis: A meta-analysis
Source: PLoS One. 2020 Oct 28;15(10):e0240251. doi: 10.1371/journal.pone.0240251 (PMC7592798; doi:10.1371/journal.pone.0240251)
Supplement: Attachment — Submitted filename: Response to Reviewers-8.3.docx [file pone.0240251.s003.docx]

Dear Editors and Reviewers:

Thank you for your letter and for the reviewers'comments concerning our manuscript entitled“The prevalence of *fimA* genotypes of Porphyromonas gingivalis in patients with chronic periodontitis: a meta-analysis” (ID: PONE-D-20-06291R1). Those comments are all valuable and very helpful for revising and improving our paper, as well as the important guiding significance to our researches. We have studied comments carefully and have made correction which we hope meet with approval. We have polished the language of the full text, see supporting information for the certificate. Revised portion are marked in red in the paper. The main corrections in the paper and the responds to the reviewer’s comments are as flowing:

Responds to the reviewer’s comments:

**Response to comment:**1. If the authors have adequately addressed your comments raised in a previous round of review and you feel that this manuscript is now acceptable for publication, you may indicate that here to bypass the “Comments to the Author” section, enter your conflict of interest statement in the “Confidential to Editor” section, and submit your "Accept" recommendation.

Reviewer #1: (No Response)

**Response:** The author has dealt with the comments raised in the last round of review. Of course, there may be insufficient answers. The author will make supplementary amendments according to the opinions of this round.

**Response to comment**:2. Is the manuscript technically sound, and do the data support the conclusions?
The manuscript must describe a technically sound piece of scientific research with data that supports the conclusions. Experiments must have been conducted rigorously, with appropriate controls, replication, and sample sizes. The conclusions must be drawn appropriately based on the data presented.

Reviewer #1: No

**Response:** This study describe a technically sound piece of scientific research with data that supports the conclusions. A total of 167 studies were identified through the search process. The articles processed were based on abstracts, titles and full texts, with a total of 17 papers were included. The total sample size was 1389 individuals with samples collected from different countries. The conclusions of this study are drawn from the data extracted from the literature.

**Response to comment:**3. Has the statistical analysis been performed appropriately and rigorously?

Reviewer #2: No

**Response:** The statistical methods used in this study have been explained in detail in the statistical analysis part of the article.
We used a statistical software package (Stata, version 11.0/mp, statacorp) to calculate the data analysis for each combined incidence estimate.

**Response to comment:**5. Is the manuscript presented in an intelligible fashion and written in standard English?
Reviewer #1: No

**Response:** The article has been revised in standard English.

Reviewer #1:

Response to comment:- This revised draft has been improved to some extent, no doubt. However, there are still minor and major drawbacks, and this re-submitted version is not considered ready to proceed.
- Again, the authors are strongly encouraged to seek help from a native speaker experienced with scientific writing.
**Response:** We are very sorry for our negligence of the minor and major drawbacks in this draft ,according that we revised the draft thoroughly. In addition, we seek the help from the native speaker to revise our draft.

**Response to comment:**- Title - "The prevalence of fimA genotype of Porphyromonas gingivalis in patients with chronic periodontitis :a meta-analysis". Please revise for correct punctuation. Remember that it is not considered the reviewers' task to co-author and/or re-edit your submitted draft.
- Go for italics with your species names.

**Response:** We are very sorry for our incorrect writing of the title. We have revised the title's punctuation and italics with species names.
**Response to comment:**- The term "prevalence of fimA genotype of Porphyromonas gingivitis" would seem misleading. Remember that you did not study other genotypes than "fimA". (Indeed, you have focussed on the fimA Ⅱ and fimA Ⅳ genotypes). Please revise thoroughly.

**Response:** Thank you for your kind reminder. We have performed the genotyping analyses for fimA I, II, III, IV and V. The results were shown in Fig. 2 through Fig. 7.
**Response to comment:** Abstract -- Not acceptable in the present form. This reviewer indeed is somewhat astonished about the low quality of this revised draft. Please see, for example:
* ( P.gingivalis) must read (P. gingivialis) (must be italicized). Remember to define abbreviations upon first appearance in the text, then use the abbreviated forms.

**Response:** We have made correction according to the Reviewer’s comments.

**Response to comment**:* Please stick to PLOS ONE's Guidelines for Authors: "The Abstract — which must be no more than 300 words long and contain no references — should serve both as a general introduction to the topic and as a brief, non-technical summary of the main results and their implications." Ignoring these recommendations must lead to outright reject, in particular with a re-submitted draft. Please note that this part must be a stand-alone section, enabling future readers to switch to your full text. Provide as much information as possible here, but do not repeat again and again multiple aspects. For example, "P. gingivitis" has been mentioned 11 (!) times.

**Response:** We have made correction according to the Reviewer’s comments.

**Response to comment:**- Double check for grammar and style.

**Response:** We have made correction according to the Reviewer’s comments.

**Response to comment:**- What is "Martials"?

**Response:** We have made correction according to the Reviewer’s comments.

**Response to comment:**- Stick to the established sections and their respective names given with the Authors' Guidelines.

**Response:** We have made correction according to the Reviewer’s comments.

**Response to comment:**- Delete blank spaces and hard paragraphs marks considered dispensable.

**Response:** We have made correction according to the Reviewer’s comments.

**Response to comment:**- In contrast, "periodontaldisease" must read "periodontal disease". Same with "fimA Ⅳand", "fica ⅤPorphyromonas", "respectively.Conclusion", fimAⅡand". Please revise thoroughly, and remember that authors are responsible for procuring copy editing or language editing services for their manuscripts. Obtaining this service is the responsibility of the author, and should be done before initial submission (but NOT after the re-submission).

**Response:** We are very sorry for our incorrect writing . We have made correction according to the Reviewer’s comments.

**Response to comment:** Intro - Same aspects as given above would be evident with the full text sections. This revised and re-submitted draft would not seem ready to proceed. Major revisions would seem mandatory.
- Please remember that each and every statement needs a reference. See, for example, "Periodontitis is a chronic inflammation (...) characterized by the progressive loss of attachment and alveolar bone absorption." Reference missing.

**Response:** We have made correction according to the Reviewer’s comments.

**Response to comment:**- Again, authors are strongly encouraged to visit a recent paper published by PLOS ONE. There you will see that referencing does follow a format like this: "(...) interdental hygiene [6, 9], even with high-risk (...) [10, 11]. In fact, this approach (...) [12]." This also is clearly stated with the Guidelines for Authors, so please revise thoroughly throughout your text.

**Response:** We have made correction according to the Reviewer’s comments.
**Response to comment:**- See last recommendation: "Again, please elaborate your aims, and give your objectives." Obviously, the authors did not want to follow here.

**Response**: We have made correction according to the Reviewer’s comments.
**Response to comment:**- See last recommendation: "Deduce a sound and indisputable null hypothesis from your foregoing elaboration." Obviously, the authors did not want to follow here.
**Response:** We have made correction according to the Reviewer’s comments.

**Response to comment:** Meths - From this revised draft it would seem clear that only two of the included studies were rated "high quality". This is considered poor.
**Response:** Thank you for your comments. Yes. There are less high-quality original articles used for the meta-analysis. So we performed the further analysis to compare the high-quality and low-quality articles.

**Response to comment:**- What about possible bias of the included studies?

**Response:** There may be publication bias, that is, there may be negative results not published.
**Response to comment:-** While some scientists occasionally have considered the systematic review (SR) as the top level of evidence for clinical science research in the past, please note that SR per se cannot be endorsed solely due to their format ("because they are a systematic review"), but must be critically evaluated for the problem reviewed in the respective article. Consequently, different qualities of RCTs, explicit statements of protocols, careful estimates of risk bias, large numbers of studies, and sophisticated statistical meta-analyses are not considered true substitutes for a thorough understanding of the literature, for critical analysis, and for scientifically sound and clinically relevant conclusions.
**Response:** Yes. I very agree with you. The meta-analysis is only the analysis of more data by collecting the related studies when there are less clear results. The aim of meta-analysis is for higher power of statistical analysis. But the results were affected by the quality of the original article. In fact, it should be best for RCT with large participants, just as the RECOVERY Study done by Oxford University for the study of COVID-19.

**Response to comment:** Results- Providing several forest plots without guiding the reader would not seem acceptable.

**Response**: We have made correction according to the Reviewer’s comments.

**Response to comment:** Disc- This section still has not been convincingly elaborated.
- Please do not simply repeat your results here. Instead, provide an intellectual discourse.
- Remember that section heading is NOT "Repetition". Provide a critical analysis of your findings. Provide interpretations. Discuss limitations.
- Please see above, and stick to the weaknesses of the included papers, give explanations and arguments.
- What about your null hypothesis?
**Response:** Considering the Reviewer’s suggestion, We have re-written this part according to the Reviewer’s suggestion.

**Response to comment:** Refs- Again, the authors obviously have failed to revise this section for a constant formatting, and this would seem surprising with a revised draft. Thorough revision still is mandatory, but, again, this would not seem the reviewer's task.

**Response:** We have made correction according to the Reviewer’s comments.

Reviewer #2:

**Response to comment:** Why didn't you adjust for factors such as smoking, alcohol , age and gender? Also some spaces between the words were ignored. Please make the correction.

**Response:** Thank you for your comments. Yes. We should adjust the smoking, alcohol , age and gender, but the data were inadequate. We have corrected the spaces between the words.

Reviewer #3:

**Response to comment**: Introduction:
1. Spacing is needed after the comma : Porphyromonas gingivalis,Aggregatibacte actinomycetemcomitans,

**Response:** We have made correction according to the Reviewer’s comments.
**Response to comment:** 2. Aggregatibacte actinomycetemcomitans: Spelling mistake: Correct would be Aggregatibacter actinomycetemcomitans
**Response:** We have made correction according to the Reviewer’s comments.

**Response to comment:**3. Term P.gingivalis has been used only in few places in the introduction but the term Porphyromonas gingivalis has been used is the most part of the manuscript. Keeping it uniform would better help the readers.
**Response:** We have made correction according to the Reviewer’s comments.

**Response to comment:** Letter A in Acknowledgements and Letter R in References needs to bold to make it uniform.

**Response:** We have made correction according to the Reviewer’s comments.

**Response to comment:**7. PLOS authors have the option to publish the peer review history of their article ([what does this mean?](https://journals.plos.org/plosone/s/editorial-and-peer-review-process#loc-peer-review-history)). If published, this will include your full peer review and any attached files.
If you choose “no”, your identity will remain anonymous but your review may still be made public.
**Do you want your identity to be public for this peer review?** For information about this choice, including consent withdrawal, please see our [Privacy Policy](https://www.plos.org/privacy-policy).

Reviewer #1: No

Reviewer #2: No

Reviewer #3: No

**Response:** We have made correction according to the Reviewer’s comments.

Thank you！
