# Supplementary material for: The prevalence of fimA genotypes of Porphyromonas gingivalis in patients with chronic periodontitis: A meta-analysis
Source: PLoS One. 2020 Oct 28;15(10):e0240251. doi: 10.1371/journal.pone.0240251 (PMC7592798; doi:10.1371/journal.pone.0240251)
Supplement: Attachment — Submitted filename: Response to Reviewers.docx [file pone.0240251.s004.docx]

**Dear Editors and reviewers:**

Thank you for your letter and for the reviewers'comments concerning our manuscript entitled "The prevalence of *fimA* genotypes of Porphyromonas gingivalis in patients with chronic periodontitis: a meta-analysis" (ID: PONE-D-20-06291R1). Those comments are all valuable and very helpful for revising and improving our paper, as well as the important guiding significance to our researches. We have studied comments carefully and have made correction which we hope meet with approval. Revised portion are marked in red in the paper. The main corrections in the paper and the responds to the reviewer’s comments are as flowing:

**Responds to the reviewer’s comments:**

1. If the authors have adequately addressed your comments raised in a previous round of review and you feel that this manuscript is now acceptable for publication, you may indicate that here to bypass the “Comments to the Author” section, enter your conflict of interest statement in the “Confidential to Editor” section, and submit your "Accept" recommendation.

Reviewer #1: (No Response)

**Response: The author has dealt with the comments raised in the last round of review. Of course, there may be insufficient answers. The author will make supplementary amendments according to the opinions of this round.**

2. Is the manuscript technically sound, and do the data support the conclusions?
The manuscript must describe a technically sound piece of scientific research with data that supports the conclusions. Experiments must have been conducted rigorously, with appropriate controls, replication, and sample sizes. The conclusions must be drawn appropriately based on the data presented.

Reviewer #1: No

**Response:** **This study describe a technically sound piece of scientific research with data that supports the conclusions. We evaluated the quality of the 17 included literatures and all met the requirements. The conclusions of this study are drawn from the data extracted from the literature.**

5. Is the manuscript presented in an intelligible fashion and written in standard English?
PLOS ONE does not copyedit accepted manuscripts, so the language in submitted articles must be clear, correct, and unambiguous. Any typographical or grammatical errors should be corrected at revision, so please note any specific errors here.

Reviewer #1: No

**Response: Thank you for your comments. We have revised our article in standard English.**

- Please change sequence: "A total of 17 studies were included in this report." should follow after "The reference lists of relevant published articles were searched manually."

**Response: Thank you for your comments. We have made correction according to the Reviewer’s comments.**

- PMID number never will appear with your text, and this reviewer clearly is wondering about the authors' intention. Again, it should be emphasized that having a closer look into recently published PLOS ONE papers would be advantageous. Please delete "( PMID:23991022 )" and all other PMIDs. These should appear with your Ref section.

**Response: Thank you for your comments.We have made correction according to the Reviewer’s comments.**

- Same with your Ref numbers. This must be separated by using the spacebar. See, for example: "(...) can lead to the loss of affected teeth [1]." Please note that this should not be necessary with a third (re-)submission, and, to be honest, the authors should re-think their attitude towards their willingness to adequately revise their draft. Remember that with your re-submission, you affirm that ALL FOUR CO-AUTHORS have read and approved this revised draft.

**Response:** **Thank you for your comments. We have made correction according to the Reviewer’s comments.**

- What do you refer to when stating "In this study, (...)"? Do you mean the present study, or do you refer to [14]? Again, please revise thoroughly, to facilitate reading.

**Response: Thank you for your comments. We have made correction according to the Reviewer’s comments.**

- Again, please elaborate both aims and objectives more clearly. Remember that aims are statements of intent. They are usually written in broad terms. They set out what you hope to achieve at the end of the project. Objectives, on the other hand, should be specific statements that define measurable outcomes, e.g. what steps will be taken to achieve the desired outcome.

**Response: Thank you for your comments. We have made correction according to the Reviewer’s comments.**

- Finally, a clear and indisputable null hypothesis is missing. Please remember that H0 must be deducible from the foregoing thoughts.

Please note that this section still would not seem convincing.
**Response: Thank you for your comments. We have made correction according to the Reviewer’s comments.**

- "All articles with the keywords were (...)." How many articles are you talking about?

**Response: Thank you for your comments. We have made correction according to the Reviewer’s comments.**

- "Studies were excluded (...)." How many articles are you talking about? Please add numbers.

**Response: Thank you for your comments. We have made correction according to the Reviewer’s comments.**

- "(...) assessment tool.[28] We assessed (...)." Why do you superscript the Ref number here?

- "The total score for each study was between 0 and 8." This would be self-explaining. What about providing a complete analysis, thus revealing the exact quality of the various studies?

**Response:** **Thank you for your comments. The purpose of marking the reference number here is to better explain to readers the basis of literature scoring through references. Of course, the author also revised table 1 to list the detailed scoring details of each literature, thus revealing the exact quality of various studies. Refer to and use effective quality evaluation standards to evaluate the quality of the included literature (scoring basis): (1 point for meeting one item)**

**(1) Clear definition of target population**

**(2) Representativeness of sampling**

**(3) The matching of samples and the whole population**

**(4) Sufficient response rate**

**(5) Standardized data collection methods**

**(6) Reliable investigation method**

**(7) Effective investigation measures**

**(8) Appropriate statistical methods**

- "(...) and therefore the results of most related studies were included in the analysis." Most, but not all, right? Again, please add complete and exact information, and do not stick to vague statements.
**Response: Thank you for your comments. We have made correction according to the Reviewer’s comments.**

- Please stick to H0 when staring this section.

**Response: Thank you for your comments. We have made correction according to the Reviewer’s comments.**

- "(...) in mouse models[31](PMID: 17081195)[36] and (...)." Please see comments given above. Make use of your spacebar to separate Refs, delete PMID, and do not superscript Refs.

**Response: Thank you for your comments. We have made correction according to the Reviewer’s comments.**

- "Presently, there is no other meta-analysis similar to this research in terms of ability to compare results." One sentence does not constitute one paragraph.

**Response: Thank you for your comments. We have made correction according to the Reviewer’s comments.**

- "First, publication bias is an inevitable problem in a meta-analysis process." This should be discussed more thoroughly. Same with the other aspects.

**Response: Thank you for your comments. We have made correction according to the Reviewer’s comments.**

- "In conclusion, P. gingivalis is one of main bacteria in chronic periodontitis." This aspect is not considered a conclusion deducible FROM YOUR STUDY.

**Response: Thank you for your comments. We have made correction according to the Reviewer’s comments.**

- Same with "These results indicate that the pathogenicity of other fimA genotypes of P. gingivalis needs to be further studied." This might be widely spread, but, to be honest, would be a simple stereotype only.

**Response: Thank you for your comments. We have made correction according to the Reviewer’s comments.**

- Please note that your Refs still have not been uniformly formatted. Again, please check Guidelines, and consult some recent papers published in PLOS ONE.

Response: **Thank you for your comments. We have made correction according to the Reviewer’s comments.**

**Thank you!**
